# The association between self-reported vaping and the human oral microbiome: a systematic review

**DOI:** 10.1093/ntr/ntag103

**Published:** 2026-05-15

**Authors:** Joseph Sidhom, Sudheer Babu Balla, Santosh Kumar Tadakamadla, Jyothi Tadakamadla

**Affiliations:** Dentistry and Oral Health, Department of Rural Clinical Sciences, La Trobe Rural Health School, 109 Arnold St, Bendigo, VIC 3550, Australia; Dentistry and Oral Health, Department of Rural Clinical Sciences, La Trobe Rural Health School, 109 Arnold St, Bendigo, VIC 3550, Australia; Violet Vines Marshman Centre for Rural Health Research, La Trobe Rural Health School, Edwards road, Flora Hill, Bendigo, 3552, Australia; Dentistry and Oral Health, Department of Rural Clinical Sciences, La Trobe Rural Health School, 109 Arnold St, Bendigo, VIC 3550, Australia; Violet Vines Marshman Centre for Rural Health Research, La Trobe Rural Health School, Edwards road, Flora Hill, Bendigo, 3552, Australia; Dentistry and Oral Health, Department of Rural Clinical Sciences, La Trobe Rural Health School, 109 Arnold St, Bendigo, VIC 3550, Australia; Violet Vines Marshman Centre for Rural Health Research, La Trobe Rural Health School, Edwards road, Flora Hill, Bendigo, 3552, Australia

## Abstract

**Introduction:**

Vapes, with or without nicotine, have experienced a rise in their popularity, and our understanding of how vapes affect our oral microbiome remains to be reviewed. This systematic review explores whether the use of vapes is associated with measurable alterations in the diversity and taxonomic composition of the oral microbiome compared to non-vapers or conventional cigarette smokers.

**Methods:**

A comprehensive and systematic search of 6 databases was meticulously conducted, covering published literature up to the end of August 2024. The data was then extracted and underwent a qualitative analysis. Studies’ risk of bias was assessed using Newcastle Ottawa Scale. Eligible studies needed to have a comparison group of either non-smokers or tobacco users or both and a group of sole e-cigarette users.

**Results:**

The search yielded 18 articles, with sixteen being cross-sectional and the remaining two were cohort studies. There were 1418 participants across the studies, ranging from 30-125 participants in each study. Majority of the included literature indicated e-cigarettes can alter the taxonomic composition and diversity of their user’s oral microbiome.

**Conclusions:**

Vaping was found to be associated with changes in oral microbiome. Since, the majority of the existing literature relied on self-reporting to see if participants were sole vapers, and mainly focused on bacterial genera, the exact impact of these changes are difficult to ascertain. However, the existing literature demonstrates a relationship in a few bacterial genera associated with periodontal disease among e-cigarette users, highlighting the need for further comprehensive studies with sound methodological approach to recruit sole e-cigarette users.

**Implications:**

This study concludes that vapes do have a demonstratable impact on the oral microbiome that is unique to vapes. E-cigarette use may be associated with an increase periodontal pathogens which may predispose e-cigarette users to worse periodontal disease, however, further research is required.

## Introduction

The global use of vapes or electronic cigarettes (EC), has been steadily increasing.^1^ These battery-operated devices function by heating a liquid to produce an aerosol, which the user then inhales.^2^ The liquid typically comprises a base of propylene glycol and vegetable glycerine, along with a range of flavoring agents, menthol, and sweeteners, depending on the specific product. In many formulations, nicotine is present, often accompanied by its derivative, nicotyrine.^3^^,^^4^ In Australia, the prevalence of individuals aged 14 and over who reported having ever used EC rose to nearly 1 in 5 in 2022–2023, a significant increase from 8.8% in 2016.^5^

Under healthy conditions, a synergistic relationship exists between the host and the diverse microbial communities residing in the oral cavity. Maintaining a balanced oral microbiome is crucial for preserving homeostasis and plays a vital role in preventing oral diseases, such as periodontitis and dental caries.^6^^,^^7^ However, several external factors, such as poor oral hygiene, infections, dietary habits, medication use, and tobacco smoking, can disrupt microbial equilibrium, leading to a state known as oral dysbiosis.^8^ Emerging evidence suggests a potential association between oral microbial imbalances and oral cancer.^6^ Importantly, the influence of the oral microbiome dysbiosis extends beyond the oral cavity, with dysbiosis implicated in systemic conditions, including cardiovascular and respiratory diseases as well as gastrointestinal cancers.^9–11^

While the effects of diet, oral hygiene, infections, medications, and traditional smoking on the oral microbiome are well-documented, the impact of EC use remains insufficiently explored.^8^^,^^12^ Traditional smoking has been found to reduce oxygen levels in the oral environment, thereby promoting the growth of bacterial genera such as Streptococcus, Prevotella, and Veillonella.^12^^,^^13^ These shifts underscore how the inhalation of smoke can profoundly alter the composition and function of the oral microbiome. Emerging in vivo studies suggest that the use of EC may also influence oral microbial communities.^14^ However, to date, no systematic review has comprehensively synthesized the clinical evidence on this topic. Guided by the PECO (Participants, Exposure, Comparator and Outcome) framework, the research question of this systematic review was, whether use of vapes (Exposure) in human participants (Participants) is associated with measurable alterations in the diversity and taxonomic composition of the oral microbiome (Outcome) compared to non-vapers or conventional cigarette smokers (Comparator).

## Methods

This systematic review conforms with the Preferred Reporting Items for Systematic Reviews and Meta-Analyses (PRISMA) guidelines.^15^ It was registered with the International Prospective Register of Systematic Reviews (PROSPERO) under the registration number CRD42024565965.

### Search strategy

The following databases were searched to identify relevant studies for this systematic review: MEDLINE, Embase, Scopus, Cochrane Database of Systematic Reviews, Web of Science, and CINAHL. Each database was searched from its inception up to October 2024. The search strategy was initially developed for MEDLINE and subsequently adapted for use in the other databases. The detailed search strategy for MEDLINE is presented in Box 1. Additionally, the reference lists of all included articles were screened to identify any further studies that met the inclusion criteria. Tables S1 to S5 in supplementary file summarizes the search strategies used in other databases. Furthermore, a search of the gray literature was conducted by searching the reference lists of all included articles and reviewing citations on relevant organizational websites such as the World Health organization, Oral Health Victoria and FDI world dental to identify any further eligible studies.

**Figure 1:**
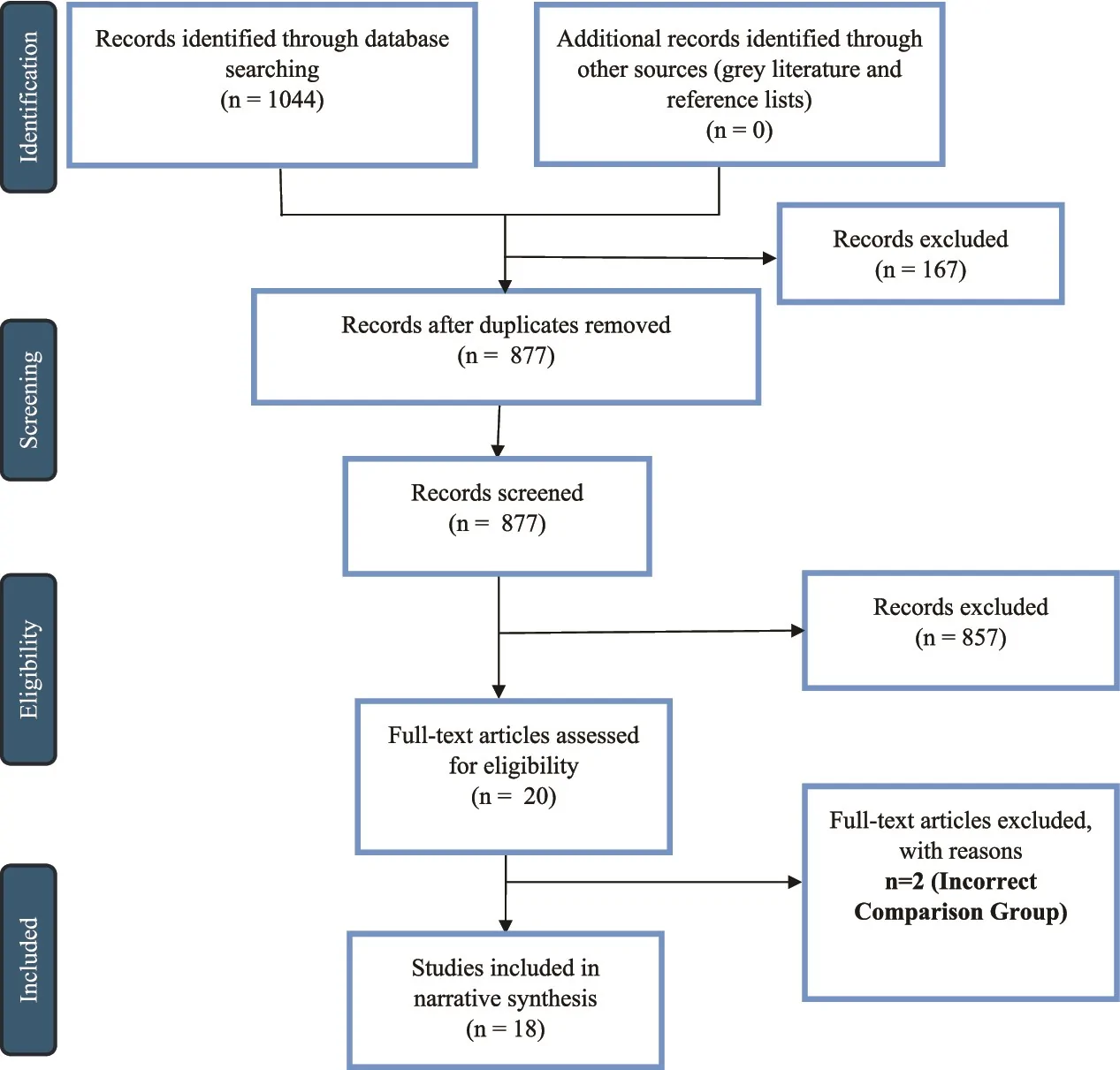
PRISMA flow chart demonstrating flow of studies. PRISMA flowchart showing the identification of 1044 studies, screening of 877, and final inclusion of 18 studies. n: Number of studies found.

**Box 1: Search strategy used in Medline**

Electronic Nicotine Delivery Systems (MeSH) OR Vaping (MeSH) OR “e-cig^*^” OR “ecig^*^” OR “electronic cigarette^*^” OR “electronic smoking device^*^” OR “Electronic Nicotine Delivery System^*^” OR “electronic non-nicotine delivery system” OR “e-vapor^*^” OR “e-vapor^*^” OR vape^*^ OR vaping OR “e-liquid^*^” OR “e-smoking” OR “e-juice^*^” OR “heated tobacco product^*^” OR “tobacco heating product^*^” OR juul^*^ AND “Oral microbiome” OR “Oral microbio^*^” OR “Oral adj2 microbio^*^” OR “Oral bacteria^*^” OR “Oral biofilm^*^” OR “biofilm” OR “microbio^*^” OR “mircobiota” OR “microbiome” OR “microflora”

**Table 1:** Changes in alpha and beta diversity among electronic cigarettes users compared to non-smokers.

	**Alpha diversity**	**Beta diversity**	**Sample collection site**
**Yang 2023^31^**	Increase	Decrease	Dorsal tongue, hard palate, buccal mucosa, and keratinized gingiva
**Stewart 2018^29^**	Negative	Negative	Saliva
**Wang 2022^30^**	Increase	Decrease	Saliva
**Park 2023^27^**	Increase	Decrease^*^	Saliva and Subgingival Sites
**Ganesan 2020^23^**	Increase	Decrease	Subgingival plaque from Maxillary First Molar
**Xu 2022^20^**	Increase	Decrease	Saliva
**Chopyk 2021^14^**	Increase^**^	Decrease	Saliva and Buccal Mucosa
**Liu 2024^25^**	Negative	Decrease	Saliva
**Pushalkar 2020^28^**	Increase	Decrease	Saliva
**Thomas 2022^19^**	Negative	Decrease	Subgingival Plaque
**Ying 2022^19^**	Negative	Negative	Saliva

^*^Positive in Subgingival but negative in salivary sample.

^^**^^Positive in salivary but negative in Buccal sample.

**Table 2:** Association of electronic cigarette use with common bacterial genera.

**Bacteria genera**	**Studies which demonstrated increase in Vapers vs NS**	**Studies which demonstrated decrease in Vapers vs NS**
**Aggregatibacter**	Thomas^19^	Chopyk^14^
**Actinomyces**	Xu,^20^ Chopyk,^15^ Pushalkar,^28^ Aldakheel^21^	Park^27^
**Prevotella**	Chopyk,^15^ Wang,^30^ Thomas,^19^ Miluna-Meldere,^35^ Yang,^31^ Pushalkar^28^	
**Dialister**	Park,^27^ Xu^20^ and Pushalkar^28^	
**Fusobacterium**	Wang,^29^ Xu,^20^ Pushalkar^28^	
**Granulicatella**	Ganesan^23^ and Chopyk^15^	Thomas^19^
**Peptostreptococcus**	Ganesan,^23^ Park^27^	Liu^25^
**Porphyromonas**	Miluna-Meldere,^35^ Wang,^30^ Pushalkar,^28^ Aldakheel^21^	Park,^27^ Thomas,^19^ Liu^25^
**Streptococcus**	Wang,^30^ Ganesan^23^	Park,^27^ Pushalkar,^28^ Thomas^19^
**Tannerella**	Thomas,^19^ Miluna-Meldere,^35^ Yang,^31^ Aldakheel^21^	
**Treponema**	Park,^27^ Xu,^20^ Aldakheel^31^	
**Veillonella**	Yang,^31^ Wang,^30^ Ganesan,^23^ Chopyk,^14^ Liu,^25^ Pushalkar^28^	

Table 2: Table showing how vapes influence certain bacterial genera, either increasing or decreasing, across the included studies.

### Eligibility criteria and selection process

All search results from the database queries and supplementary sources were exported into Covidence,^16^ where duplicates were removed automatically. Two reviewers (JS and SBB) then independently screened the titles and abstracts; any disagreements were resolved through discussion until a consensus was reached. Articles judged potentially relevant underwent full-text review by the same reviewers, with unresolved discrepancies adjudicated by a third reviewer (JT). Eligible study designs included randomized control trials, cross-sectional, cohort, case–control, and non-randomized clinical trials. Eligible studies needed to have a comparison group of either non-smokers or tobacco users or both and a group of sole e-cigarette users. Conference abstracts, case reports, studies involving non-human participants and those published in a language other than English were excluded.

### Data extraction

Data from each included study were extracted into a pre-piloted Microsoft Excel spreadsheet. The following information was extracted from the included studies, including the lead author, publication year, study design, as well as sample characteristics such as specimen type, participant age range, and definitions of comparison groups. Primary and secondary outcomes were documented in the same spreadsheet. Microbiological results were captured in detail, including measures of α-diversity (number of observed species and evenness of abundance within subjects) and β-diversity (measured dissimilarity of bacterial communities between groups), method of microbial assessment as well as any reported shifts in specific bacterial taxa.^17^ After extraction, studies that reported comparable α- or β-diversity metrics were grouped, and their findings were synthesized narratively. Bacterial taxa cited were also collated to identify recurring patterns.

### Data synthesis

Once extracted, the following data was presented in a table; whether alpha and beta diversity increased or decreased, bacterial taxa that the studies stated were influenced by EC usage and the direction of association was also recorded. The effect of secondary in-vivo outcomes and how the microbial shifts in vapers compared to that in tradition cigarette users was also recorded. Bacterial taxa that were demonstrated to be influenced by EC use in at least two studies and what the studies reported as EC’s influence on these bacterial taxa was recorded in a separate table. A summary of the alpha and beta diversity findings was also presented in another table for clarity. Due to substantial heterogeneity in the reported outcomes, a meta-analysis was deemed inappropriate, and a qualitative synthesis was instead undertaken.

### Risk of bias assessment

Studies meeting the inclusion criteria were assessed using the Newcastle–Ottawa Scale (NOS).^18^ NOS comprises three domains: Selection (maximum of 4 stars), Comparability (maximum of 2 stars), and Exposure (maximum of 3 stars). The Selection domain evaluated case definition, representativeness of cases, selection of controls, and definition of controls; the Comparability domain examined how well studies controlled key confounders, such as periodontal status and oral hygiene practices; and the Exposure domain assessed ascertainment of exposure, consistency of ascertainment methods across groups, and non-response rates. For cohort studies, the wording of the NOS was adapted to capture cohort-specific sources of bias, such as age, gender and overall systemic health, while retaining the same three-domain structure and scoring system.^18^ Overall study quality was categorized as good when studies achieved a total of 7 to 9 stars, fair when they scored 4-6 stars and poor when they scored a total of 3 or below. The first reviewer (JS) conducted the initial quality assessment for each study. The findings were then compared with the independent appraisal of the second reviewer (SBB). Any discrepancies between the reviewers were resolved through discussion.

## Results

The flow of study selection is summarized in Figure 1.

The initial search identified 1045 articles. After removing duplicates, 877 articles were screened based on their titles and abstracts. Of these, 20 studies were selected for full-text review, and 18 met the inclusion criteria for the final analysis.^14^^,^^19–36^ Collectively, these studies involved 1418 participants, including 456 exclusive EC users, with individual study sample sizes ranging from 30 to 125 participants.

### Study characteristics

The majority of the studies (*n* = 16) employed a cross-sectional design,^14^^,^^21–36^ while two studies, conducted by Thomas et al.^19^ and Xu et al.^20^ were cohort studies with a six-month recall period.

Twelve of the included studies assessed beta-diversity, alpha-diversity, and relative abundance of specific bacterial taxa (RA).^14^^,^^19^^,^^20^^,^^25^^,^^27–31^^,^^34^ One study evaluated microbial load using colony-forming units.^22^ In contrast, three studies focused on RA as an outcome measure.^22^^,^^26^^,^^33^ Additionally, one study employed descriptive statistics due to its limited sample size,^35^ and another utilized an enzymatic method to quantify dysbiosis.^24^ (Tables S6 and S7).

### Study findings

#### Diversity compared to never smokers

Four studies reported no significant changes in the alpha diversity of the oral microbiome in EC users when compared to non-smokers.^14^^,^^19^^,^^25^^,^^29^ Among these, the study by Chopyk et al.^14^ noted an increase in alpha diversity specifically in saliva samples, with no corresponding change observed in the buccal microbiome (Table 1). In terms of beta diversity, most studies found a decrease among e-cigarette users relative to both non-smokers and traditional cigarette (TS) smokers (Table 1). However, two studies^27^^,^^29^ diverged from this trend. Park et al.^27^ reported no changes in beta diversity within the salivary microbiome but did observe decreases in the subgingival microbiome. Meanwhile, Stewart et al.^29^ and Ying et al.^34^ were the only studies that found no significant changes in either alpha or beta diversity and also reported no alterations in the taxonomic relative abundance of bacterial species. (Table S7).

In contrast to diversity-based analyzes, the study by Kiiun^24^ adopted an enzymatic approach to assess dysbiosis levels. Their findings indicated that e-cigarette use was associated with an increased degree of oral dysbiosis.

#### Taxonomic characteristics compared to never smokers

Although not all studies investigated both alpha and beta diversity, nearly all examined the effects of EC use on at least one bacterial species. Among studies focusing on the subgingival microbiome, a consistent shift in microbial composition was observed, with an increase in anaerobic bacteria such as *Treponema denticola*, *Tannerella forsythia*, *Veillonella*, and *Fusobacteria* (Table 2).^19^^,^^21^^,^^23^^,^^27^ Similarly, studies assessing the salivary microbiome, excluding those by Stewart et al.^29^ and Ying et al.^34^ reported an increased abundance of *Veillonella* and *Prevotella* species among EC users (Table 2).^14^^,^^20^^,^^28^^,^^30^^,^^35^ However, findings were inconsistent regarding the effects of vaping on *Porphyromonas* levels, with some studies reporting increases.^21^^,^^28^^,^^30^^,^^35^ In contrast, others found no significant change.^19^^,^^21^^,^^25^^,^^27^^,^^28^^,^^30^ (Table S5).

Research on the buccal microbiome revealed outcomes comparable to those observed in salivary studies, suggesting similar microbial shifts.^14^^,^^31^^,^^33^ One study by Alzoubi et al.^22^ focused solely on *Viridans streptococci* and found no effect from e-cigarette use. Across all studies that assessed, *Streptococcus mutans, this species* was either unaffected or showed a decrease in prevalence among EC users.^19^^,^^27^^,^^28^ The impact of vaping on *Candida* carriage was addressed in only three studies, Alzoubi et al.^22^ Mokeem et al.^26^ and Cichonska et al.^33^ While Alzoubi et al.^22^ and Cichonska et al.^33^ reported no significant changes, Mokeem et al.^26^ observed an increased *Candida* carriage among vapers (Table S7).

Kiiun et al.^24^ did not do a microbial analysis to ascertain the changes in the oral microbiome caused by vaping. Instead, they used an enzymatic approach to determine the degree of dysbiosis in EC users. They concluded that e-cigarette use does increase the degree of dysbiosis.

#### Effect compared to traditional cigarettes

Twelve of the included studies made direct comparisons between the effects of EC on the oral microbiome and cigarette smoking.^19–22^^,^^25^^,^^26^^,^^28–30^^,^^33^^,^^34^^,^^36^ The majority of these studies found that, like EC use, smoking has generally been demonstrated to increase alpha diversity and decrease beta-diversity, albeit, to different and often greater levels.^19^^,^^21^^,^^25^^,^^26^^,^^29^^,^^30^^,^^33–36^ Only three studies found that ECs caused changes to the oral microbiome to a similar extent as traditional cigarettes.^20^^,^^22^^,^^28^ Even the nature of the shifts caused by these two systems was often unique to each other with each affecting different bacterial species differently.^22^

### Risk of bias

The Newcastle–Ottawa Scale was employed to assess the risk of bias in both the cross-sectional and cohort studies. Ten received a score greater than or equal to seven, suggesting a low risk of bias. Seven studies scored below seven and above three, indicating a moderate risk of bias. One particular study was rated a two, reflecting a high risk of bias due to inadequate control of confounding variables and poorly defined case and control groups.^36^ A detailed summary of these assessments is provided in Tables S8 and S9.

## Discussion

This systematic review aimed to understand the effects of vapes or EC on the human oral microbiome. However, methodological heterogeneity between the included studies makes it difficult to draw definitive conclusions, particularly regarding their effects on alpha diversity. Studies often did not control for certain characteristics such as duration and frequency of EC use. In addition, participants’ periodontal status was not always consistent between studies, with some examining periodontal patients and others examining patients with a healthy periodontium. Furthermore, findings on the oral microbiome according to vape composition (e.g., nicotine-containing versus nicotine-free products) could not be presented because only a limited number of studies reported the composition of the vaping liquid. Similarly, not all studies recorded if the ECs contained nicotine or had flavorings, which are factors that in vitro studies have demonstrated to have an impact on oral microbiome composition.^37^^,^^38^ This gap in reporting is likely attributable, at least in part, to limited transparency from EC producers regarding the ingredients used in their products.^39^ Despite the methodological variation, the data extracted from the included studies revealed various patterns that can provide insights into how EC use could influence the oral microbiome.

While the alpha diversity findings from the studies varied highly between studies, beta diversity findings were homogenous between studies, with studies demonstrating a decrease in beta diversity. This decrease in beta-diversity, compared to non-users of EC indicates that the samples collected from EC users had greater similarity between microbial communities when compared to non-users. Traditional cigarettes also impact beta-diversity in a similar manner.^40^ This demonstrates EC use may be associated with dysbiosis in the oral microbiome, potentially increasing the likelihood of various oral and systemic conditions. This idea is further supported by the increase in inflammatory markers in the saliva of EC users.^19^^,^^23^^,^^26^^,^^27^

The findings from the taxonomical analysis also revealed some consistent findings among the studies. Three bacterial genera, *Veillonella, Treponema, and Tannerella*, consistently showed an increased abundance across all studies which investigated them^14^^,^^19^^,^^20^^,^^22^^,^^23^^,^^26–28^^,^^30^^,^^31^^,^^35^  *Veillonella* is a gram-negative anaerobic coccus bacterium^25^ which plays a crucial role in biofilm formation, and has been most extensively studied, with six of the studies,^14^^,^^23^^,^^25^^,^^28^^,^^30^^,^^31^ unanimously reporting higher amounts in EC users compared to both traditional smokers and non-smokers. While *Veillonella’s* role in caries development is still subject to debate, as it interacts with *S. mutans*, it breaks down potent acids into weaker forms, in fact it is even found in higher amounts in patients without caries,^41^ some species of *Veillonella* such as *V. parvula* may contribute to caries.^42^ Overall, its role in caries development is complex, with some species playing a potentially protective role while others may be a risk factor for caries initiation.^43^  *Veillonella* is frequently found in early biofilm formation, with its role varying from site to site, it is a commensal bacterium and can act as a bridging species to the creation of a symbiotic microbiome,^43^ however, it can in some cases contribute as an accessory pathogen to periodontitis, but this is debated.^44^

Increase in *Tannerella* and *Treponema* in the subgingival sites of EC users was consistent across studies when compared to never smokers.^19–21^^,^^27^^,^^31^^,^^35^ The species *Tannerella Forsythia* and *Treponema Denticola* are critical components of the “red complex” in periodontitis pathogenesis, and even at low concentrations, these gram-negative anaerobes can trigger inflammatory diseases by shifting the oral microbiome into a state of dysbiosis.^40^ However, it is important to note that only three studies^19^^,^^21^^,^^35^ specified the species *T. forsythia* as being increased*, T. denticola* was not specifically mentioned to be increased, only its genus *Treponema.* Not all species of these two bacteria genera play a role in periodontitis. Furthermore, most included studies reported increased *Actinomyces*^14^^,^^20^^,^^21^^,^^23^ and *Prevotella*.^14^^,^^20^^,^^23^^,^^30^^,^^31^^,^^35^ Some species of *Prevotella* have been found to be associated with periodontitis, however, others are not.^45^^,^^46^  *Actinomyces* is an early colonizer, and similar amounts can be found in carious lesions and sound tooth structure as well, so is unlikely to be a driving bacterial genera for caries.^47^ Studies which examined the subgingival microbiome controlled potential confounders such as periodontal status and oral hygiene, indicating that there is a high chance of microbial changes being attributable to the use of EC use.

The effect of EC use on other microbial residents exhibited greater heterogeneity, making it challenging to draw conclusions, particularly *Streptococcus* and *Candida*. For instance, two studies found an increase in levels of *Streptococcus* while two other found decreased levels. This uncertainty stands in contrast to in-vitro findings, which found that the flavoring in vapes creates a favorable environment for the growth of *Streptococcus*.^37^^,^^38^ The variability between our review and past studies may be due to the differences in the composition of the liquid used by participants in our included studies. Similarly, no conclusions can be drawn regarding how EC use affect Candida carriage.^23^^,^^27^^,^^34^^,^^35^ The variation in findings may be due to differences in EC use frequency, duration, user age, and composition of e-liquids across studies.

These changes to the oral microbiome in EC users may have potential implications beyond periodontal disease. *T. forsythia, Actinomyces,* and *Prevotella* have all been linked to atherosclerotic plaque formation.^48^^,^^49^ Metabolites from fusobacterium, which have been demonstrated to be increased in EC users,^20^^,^^28^^,^^30^ have been implicated in the pathogenesis of colorectal cancer and have a strong association with oral squamous cell carcinoma (OSCC). Multiple studies have reported that *Fusobacterium nucleatum* is significantly enriched in OSCC tumor sites,^50^^,^^51^ where it appears to alter anti-tumor responses and promotes epithelial-mesenchymal transition in oral cancer cells.^51^  *Treponema* has also been demonstrated to play a role in OSCC development.^52^^,^^53^ However, it needs to be understood that these are multifactorial conditions, with several aetiological and risk factors playing their part in the causation of the conditions.

When changes in the oral microbiome are compared to traditional smokers, the included studies demonstrated that the increase in these bacteria among EC users was either comparable or less pronounced.^19–22^^,^^25^^,^^26^^,^^28–30^^,^^33^^,^^34^^,^^36^ There were some similarities between the oral microbiome of conventional smokers and EC users with *Prevotella* and *Veillonella* being identified as being increased in both groups,^40^ which suggests that there may be some potential similar mechanisms in how these two products influence the oral microbiome. One such possible mechanism is the creation of an anaerobic environment in the oral cavity^48^ with Cichonska et al.^33^ demonstrating that there is an increase in anaerobes in the oral microbiome of EC users. However, it appears that EC create a unique oral microbiome, different to that of traditional smoking, suggesting that there are additional mechanisms contributing to these microbial shifts, such as the flavorings used with EC.^37^^,^^38^ Another alternative mechanism may involve the ability of certain oral bacteria to metabolize propylene glycol, which is one of the main constituents in most e-liquids.^23^ Furthermore, propylene glycol has been demonstrated to cause xerostomia, with its drying effect being further strengthened by the presence of nicotine. Xerostomia is known to contribute to oral dysbiosis.^48^

When comparing the effects of traditional smoking and vapes, a major limitation in the existing literature is highlighted. While we specifically included only those studies where the oral microbiome of exclusive e-cigarette users was studied, the majority of the included studies relied solely on self-reporting to verify patient’s use of e-cigarettes or cigarettes, which is a significant limitation. This method may not be reliable, as one of the studies which estimated Carbon Monoxide (CO) levels in participants, it was found that the participants who identified themselves as sole EC users had CO levels like that of cigarette smokers.^19^ Another study reported of using CO testing to confirm the status of participants; however, they did not report the results of their tests.^27^ Consequently, it is difficult to confirm that all the participants in the included studies, who identified as sole EC users, truly were sole EC users. Therefore, it is difficult to draw definitive conclusions from the existing body of evidence on how EC use affect specific bacterial species. However, on the level of diversity, the consistency in the findings demonstrates that EC use could be significantly associated with changes in oral microbiome.^19^^,^^21^^,^^25^^,^^26^^,^^29^^,^^30^^,^^33^^,^^34^^,^^36^

As alluded to earlier, further limitations do exist such as the heterogeneity across included studies in relation to lack of standardization in EC devices and e-liquids, limited long-term effect studies, and insufficient information on vaping patterns. Another limitation is that most of the included studies reported increases in bacterial genera, not specific species Thus, it is difficult to properly understand the effect the changes in genera would have on specific bacterial species and their subsequent impact on oral and systemic health. Furthermore, not all studies have used advanced sequence-based techniques and there is a significant heterogeneity between the studies. For this reason, a meta-analysis was not conducted due to methodological heterogeneity between the studies. However, as the literature on this topic is sparse, there are still valuable findings to present on specific bacterial species and genera that were investigated in these studies, but not on overall diversity.

Future studies should focus on recording a range of parameters associated with EC, control for confounding variables to better elucidate how these devices impact the oral microbiome. Efforts should be given to investigating how different e-liquid compositions, especially varying nicotine concentrations and flavoring agents, influence cariogenic and periodontopathic bacteria. Furthermore, future studies must focus on biologically verifying participants’ smoking status to avoid falsely classifying past or current cigarette users as sole EC users.

## Conclusion

This review concludes that EC use is associated with oral microbiome changes, as demonstrated by the decrease in beta diversity in most of the included studies. In addition, specific bacterial genera including Treponema and Tannerella, that are associated with periodontal disease, were found to be related to e-cigarette use, albeit to a similar or lesser extent than in traditional cigarette users. Despite these findings, important methodological limitations warrant consideration. The predominance of self-reported exposure data, the scarcity of long-term longitudinal studies, and insufficient characterization of e-liquid composition constrain the strength of current evidence. In particular, many studies did not clearly report whether the e-cigarettes used contained nicotine, limiting the ability to determine whether the observed microbiome changes were attributable to nicotine-containing or nicotine-free products. Although it is likely that many participants used nicotine-containing e-cigarettes, this could not be confirmed from the available data. Addressing these gaps through rigorously designed prospective studies is critical to advancing understanding of the effects of e-cigarettes on oral ecology and related health outcomes.

## Supplementary Material

Supplementary_Tables_ntag103

## Data Availability

The data underlying this article are available in the article and in its online supplementary material.
