# Supplementary material for: The association between self-reported vaping and the human oral microbiome: a systematic review
Source: Nicotine Tob Res. 2026 May 15;28(9):1480–8. doi: 10.1093/ntr/ntag103 (PMC13500349; doi:10.1093/ntr/ntag103)
Supplement: Supplementary file 1 [file supplementary_tables_ntag103.docx]

**Supplementary Information**

**Table S1: Search strategy used in EMBASE database**

exp electronic cigarette/ (MeSH) or exp vaping/ (MeSH) OR "e-cig*" or "ecig*" or "electronic cigarette*" or "electronic smoking device*" or "Electronic Nicotine Delivery System*" or "electronic non-nicotine delivery system" or "e-vapor*" or "e-vapour*" or vape* or vaping or "e-liquid*" or "e-smoking" or "e-juice*" or "heated tobacco product*" or "tobacco heating product*" or juul AND 'Oral microbiome' or 'oral microbio*' or 'oral adj2 microbio*' or 'oral bacteria*' or 'oral biofilm*' or 'biofilm' or 'microbio*' or 'mircobiota' or 'microbiome' or 'microflora'

**Table S2: Search strategy used in CINAHL database**

(((MM "Vaping")) OR (e-cig*" or "ecig*" or "electronic cigarette*" or "electronic smoking device*" or "Electronic Nicotine Delivery System*" or "e-vapor*" or "e-vapour*" or vape* or vaping or "e-liquid*" or "e-smoking" or "e-juice*" or "heated tobacco product*" or "tobacco heating product* OR juul*)) AND ('Oral microbiome' OR 'oral microbio*' OR 'oral adj2 microbio*' OR 'oral bacteria*' OR 'oral biofilm*' OR 'biofilm' OR 'microbio*' OR 'mircobiota' OR 'microbiome' OR 'microflora')

**Table S3: Search strategy adopted for SCOPUS database**

"e-cig*" OR "ecig*" OR "electronic cigarette*" OR "electronic smoking device*" OR "Electronic Nicotine Delivery System*" OR "e-vapor*" OR "e-vapour*" OR vape* OR vaping OR "e-liquid*" OR "e-smoking" OR "e-juice*" OR "heated tobacco product*" OR "tobacco heating product*" OR juul* AND ( biofilm OR microbio* OR mircobiota OR microbiome OR microflora ) W/2 oral OR ( biofilm OR microbio* OR mircobiota OR microbiome OR microflora ) W/2 saliva* "Oral microbiome" OR "oral microbio*" OR "oral microbio*" OR "oral bacteria*" OR "oral biofilm*" OR "biofilm" OR "microbio*" OR "mircobiota" OR "microbiome" OR "microflora"

**Table S4: Search strategy used in COCHRANE database**

[Vaping] MeSH descriptor OR [Electronic Nicotine Delivery Systems] MeSH descriptor explode all trees OR e-cig* or ecig* or electronic NEXT cigarette* or electronic NEXT smoking NEXT device* or Electronic Nicotine Delivery NEXT System* or ENDs or Electronic Non-Nicotine Delivery NEXT System* or ENNDs or e-vapor* or e-vapour* or vape* or vaping or e-liquid* or e-smoking or e-juice* or heated tobacco NEXT product* or tobacco heating NEXT product* or juul* AND "Oral microbiome" or oral NEXT microbio* or oral NEXT bacteria* or oral NEXT microflora* or microbiome or microbio* or microflora or "oral biofilm" or "oral biofilms

**Table S5: Search strategy administered in WEB OF SCIENCE database**

Electronic Nicotine Delivery Systems (MeSH) OR Vaping (MeSH) OR "e-cig*" OR "ecig*" OR "electronic cigarette*" OR "electronic smoking device*" OR "Electronic Nicotine Delivery System*" OR "electronic non-nicotine delivery system" OR "e-vapOR*" OR "e-vapour*" OR vape* OR vaping OR "e-liquid*" OR "e-smoking" OR "e-juice*" OR "heated tobacco product*" OR "tobacco heating product*" OR juul* AND 'ORal microbiome' OR 'ORal microbio*' OR 'ORal adj2 microbio*' OR 'ORal bacteria*' OR 'Oral biofilm*' OR 'biofilm' OR 'microbio*' OR 'mircobiota' OR 'microbiome' OR 'microflora'

**Table S6: Background characteristics and methods used in the included studies**

| **Author and Year** | **Study Design** | **Follow up Period** | **Sample Size** | **EC Users** | **TS Users** | **NS** | **Other Comparison groups** | **Area of Sample collection for microbial analysis** | **Statistical adjustments** | **Primary Outcome** | **Secondary Outcome** | **Microbe analysis** |
| --- | --- | --- | --- | --- | --- | --- | --- | --- | --- | --- | --- | --- |
| Mokheem 2018 | Cross-sectional | None | 129 | 30 | 34 | 32 | 33 | Saliva | adjusted for dual use, edentulous individuals, and systemic disorders such as acquired immune deficiency syndrome (AIDS),  cardiovascular disorders, and diabetes mellitus (DM) | Carriage of Candida | None | 18S rDNA  and 28S rDNA  primer ITS1 and ITS2 |
| Stewart 2018 | Cross-sectional | None | 30 | 10 | 10 | 10 | 0 | Buccal Swabs and Saliva | Nil Adjustements | Alpha and Beta Diversity | Taxonomic Relative Abundance | 16S rRNA  gene sequencing V4 |
| Aldakheel et al., 2020 | Cross-sectional | None | 60 | 15 | 15 | 15 | 15 (NS with perio) | 1st md molar supragingival and subgingival | Adjusted for Sample size and power, excluded based off periodontal status, systemic diseases, antibiotic use | Changes in Colony Forming Units | Nil | culture‑based  quantification of selected bacteria |
| Alzoubi 2020 | Cross-sectional | None | 90 | 30 | 30 | 30 |  | Buccal Swabs | Adjusted for antibiotic use, recent hospital admission, previous positive history of MRSA  colonisation or infection and current mouth or Upper Respiratory  Tract Infection (URTI) or ulcers such as pharyngitis, gingivitis and  herpetic ulcers. | Changes in Relative Taxonomic Units | Antibiotic resistance of microbes in E-cigarette users | Culture‑Based  Identification |
| Ganesan 2020 | Cross-sectional | None | 123 | 20 | 25 | 25 | Former TS 25  Dual EC and TS users 28 | Subgingival plaque from mx first molars | Adjusted for uncontrolled diabetes, HIV infection, use of immuno suppressant medications, bisphosphonates, or steroids, antibiotic  therapy or oral prophylactic procedures within the preceding 3 months, and  fewer than 20 teeth in the dentition | Alpha and Beta Diversity | Changes Biofilm Architecture changes (in-vitro) Taxonomical Relative Abundance (In-vivo) and Inflammatory markers (In-vivo) | Shotgun  metagenomic  sequencing |
| Pushalkar 2020 | Cross-sectional | None | 119 | 40 | 40 | 39 | 0 | Saliva | Adjusted for age, periodontal status and frequency | Alpha and Beta Diversity | Taxanomic Relative abundance | 16S rRNA  gene sequencing V3-V4 region |
| Chichonska 2021 | Cross-sectional | None | 90 | 40 | 43 | 42 | NA | Buccal Swabs | Adjusted for Smokeless-tobacco product users, Habitual alcohol users, Systemic diseases, Medication use (antibiotics, probiotics, steroids, non-steroidal anti-inflammatory drugs, bisphosphonates), Periodontal therapy (surgical or non-surgical) | Changes in Relative Taxonomic Units | None | Culture‑based  Identification |
| Chopyk 2021 | Cross-sectional | None | 24 | 12 | 0 | 12 | 0 | Buccal and Saliva | Adjusted for age, illicit drug use and frequency of smoking | Alpha and beta Diversity | Taxonomic Relative Abundance, Nasal microbiome abundance | 16S rRNA  gene sequencing V3-V4 |
| Ying 2022 | cross-sectional study | None | 28 | 10 | 8 | 10 | 0 | saliva | Adjusted for gender and age | Alpha and Beta Diversity | Taxonomical Analysis | Metatranscriptomic  RNA sequencing |
| Thomas 2022 | Cohort Study | six months | 84 | 28 | 27 | 29 | 0 | Subgingival Plaque | adjusted for periodontal status, age, medical conditions, preganancy, head and neck radioherapy, dental visit and presence of oral mucosal lesions, such as leukoplakia, herpes labialis, and candidiasis. | Alpha and Beta Diversity | Taxanomic Relative abundance | 16S rRNA  gene sequencing V3-V4 |
| Xu 2022 | Cohort Study | Six months | 101 | 32 | 31 | 38 | (22 Severe* Cigarette Smokers (SCS), 15 Severe Electronic Cigarette (SES) smokers , 11 Severe Never Smokers(SNS)) and (9 Moderate* Cigarette Smokers (MCS), 17 Moderate Electronic Cigarette smokers (MES), 27 Moderate Never Smokers(MNS)) | Saliva | Adjusted for periodontal status | Alpha and beta Diversity | Taxonomic Relative Abundance | 16S rRNA  gene sequencing V3-V4 |
| Tishchenko 2022 | Cross-sectional | None | 65 | 20 |  | 20 | 25 | Cervical region of tooth | Not adjusted | Colony Forming Units | None | Culture‑based  Identification |
| Wang 2022 | cross-sectional | None | 33 | 5 | 14 | 6 | 8 | Saliva | adjusted for sex, age, diet, height/weight, or race and duration of vape use | Alpha and Beta Diversity | Taxonomic Relative Abundance | 16S rRNA  gene sequencing  V4-V5 |
| Yang 2023 | Cross-sectional | None | 36 | 18 | - | 18 |  | dorsal tongue, hard palate, buccal mucosa, and keratinized gingiva | Not adjusted | Alpha and Beta Diversity | Relative Taxonomic Abundance | 16S rRNA  gene sequencing  V4 |
| Kiiun 2023 | Cross-sectional | None | 90 | 35 (With gingivitis) | 0 | 25 | 30 (Never smokers with gingivitis) | Saliva | Adjusted for Individuals with systemic diseases, Individuals with dental diseases, Individuals with bad habits (e.g., smoking regular cigarettes), Individuals with somatic diseases | Degree of dysbiosis units | Presence Inflammatory markers in periondontal patients and e-cigarette users | enzymatic method |
| Park 2023 | cross-sectional | none | 150 | 75 | 0 | 75 | 0 | Saliva and Subgingival Sites | Adjusted for age, gender, and the frequency  of brushing teeth | Alpha and Beta Diversity | Gingival Inflammation | 16S rRNA  gene sequencing  V3-V4 |
| Miluna-Meldere 2024 | Cohort Study | None | 31 | 9 | 4 | 5 | 13 | Saliva | Adjusted for alcohol, diet, oral hygiene, antibiotic use and systemic health conditions. | Taxanomic Relative Abundance | None | Unique PCR primers for  specific bacteria |
| Liu 2024 | Cross-sectional | None | 113 | 27 | 46 | 40 | 0 | Saliva | adjusted for chronic dry mouth, periodontal status, untreated cavity caries lesions or oral abscesses, pre-cancerous or cancerous lesions, candidiasis, halitosis, oral hygiene | Alpha and Beta Diversity | Taxanomic Relative abundance | 16S rRNA  gene sequencing  V3-V4 |

**NS:** Never Smokers
**TS:** Traditional Smokers
**EC:** Electronic Cigarette
**Perio:** Periodontitis
*Severe and moderate refer to periodontal status of participants

**Table S7: Findings related or oral microbiome from the included studies**

| **Study** | **Effect on Beta Diversity** | **Effect on Alpha Diversity** | **Taxonomical Findings compared to Never Smokers** | **Other Findings** | **EC Compared to TS** | **Verification of Smoking status** |
| --- | --- | --- | --- | --- | --- | --- |
| Mokheem 2018 | No information provided | No information provided | Increase in Candida Carriage in Vapers Compared to NS | Nil | TS users had higher Candida than EC | Self-administered questionnaire |
| Stewart 2018 | No changes | No changes | No changes | Nil | TS caused greater microbial shifts | Self-administered questionnaire and CO testing |
| Aldakheel 2020 | No information provided | No information provided | Increases in A. actinomycetemcomitans, P. gingivalis, T. denticola, T. forsythia | Nil | TS caused greater microbial shifts | Self-reporting |
| Alzoubi 2020 | No information provided | No information provided | No S.Viridans and Candida carriage in e-cig smokers. These are the only phyla examined | Higher amounts of antibiotic resistant bacteria in EC users | TS caused greater microbial shifts | Self-reporting |
| Ganesan 2020 | Increase | Decrease | Increases in Abiotrophia, Aggregatibacter, Eikenella, Granulicatella, Cardiobacterium, Hemophilus, Johnsenella, Kingella, Lachoanaerobaculum, Lautropia, Leptotrichia, Mogibacterium, Ottowia, Parvimonas, Peptostreptococcus, Rothia, Rhodobacter, Selenomonas, and Veillonella. | Higher Inflammatory markers | Similar to TS | Self-reporting |
| Pushalkar 2020 | Increase | Decrease | **Dominant Phyla:** Proteobacteria and Actinobacteria were elevated.  **Enriched Genera:** *Porphyromonas*, *Veillonella*, and *Actinomyces*. Other prominent genera included *TM7*, *Neisseria*, *Fusobacterium*, *Veillonella dispar*, *Porphyromonas endodontalis*, *Fusobacterium nucleatum subsp. vincentii*, *Prevotella oris*, *Parvimonas micra*, *P. gingivalis*, *Alloprevotella tannerae*, *Dialister invisus*, *Corynebacterium durum*, and *Leptotrichia wadei*.  **Depleted Taxa:** Included *Streptococcus oralis subsp. tigurinus clade 071*, *Porphyromonas pasteri*, *Fusobacterium periodonticum*, and *Oribacterium parvum* | Nil | Similar to TS | CO testing and Salivary Cotinine Test and Self-reporting |
| Chichonska 2021 | No information provided | No information provided | No changes to commensals More Gram negative: Staphylococcus aureus and More Gram positive: Escherichia coli, Klebsiella pneumonia No changes to Candida | Nil | TS caused greater microbial shifts | Self-reporting |
| Chopyk 2021 | Decrease in buccal no changes in saliva | Decrease | **buccal samples, increases** were observed that the relative abundance of *Haemophilus*, *Rothia*, *Veillonella*, *Actinomyces*, *Solobacterium*, *Granulicatella*, *Alloprevotella*, and *Aggregatibacter compared to NS* ***saliva of e-cigarette increases*** *in with Selenomonas, Megasphaera, Candidatus Saccharibacteria UB2523, Faucicola, Phocaeicola, Streptobacillus, Rikenellaceae RC9 group, Clostridiales group 148, and Peptoanaerobacter compared to NS* | Nil | Not reported | Self-reporting |
| Ying 2022 | No changes | Decrease | Common taxa found in EC users included:   - *Neisseria elongata* - *Neisseria sicca* - *Haemophilus parainfluenzae* | Nil | TS caused greater microbial shifts | Self-reporting |
| Thomas 2022 | No changes | Decrease | Decrease: Streptococcus, Kingella, and Scardovia, Aggregatibacter, Granulicatella, and Fretibacterium (compared to NS) Increase: Fusobacterium, Tannerella, Oribacterium, and Bacteroidales, Leptotrichia and Lactobacillus, Corynebacterium. (Compared to NS) | Nil | TS caused greater microbial shifts | Self-reporting, CO testing |
| Xu 2022 | Increase | Decrease | Enriched Filifactor and Treponema taxa in severe periodontal patients Enriched Fusobacteria taxa in the MCS and MES groups. No significant changes overtime | Nil | Similar shifts to TS | Self-reporting |
| Tishchenko 2022 | No information provided | No information provided | **Decreases S.Mitis and Increases S.Pneumoniae and S. pyogenes, No effect of Candida In both groups** (compared to NS) | Nil | TS caused greater microbial shifts | Not reported |
| Wang 2022 | Increase | Decrease | **Increases in Genus: Neisseria, Prevotellaceae, Fusobacterium, Veillonella, Streptococcus and Porphyromonas** (Compared to NS) | Nil | TS caused greater microbial shifts | Self-reporting |
| Yang 2023 | Increase | Decrease | Unspecified species of Veillonella found to be higher in exclusive EC. | Nil | No comparison | Self-reporting |
| Kiiun 2023 | No information provided | No information provided | No information provided | Vapers had higher dysbiosis units compared to non-vapers and inflammatory markers | No Comparison | Self-reporting |
| Park 2023 | Increase | Decrease only in subgingival microbiome, not in saliva | **Decreases in Microbial Abundance**   - **Both Subgingival and Saliva Sites:** A reduction in the relative abundance of *Bergeyella*, *Neisseria*, *Enterococcus*, and *Haemophilus* was detected in both subgingival plaque and salivary samples. - **Saliva Only:** A decrease in the abundance of *Catonella* was specifically observed within the saliva. - **Subgingival Sites Only:** A reduction in the relative abundance of the following genera was confined to the subgingival microenvironment: *Porphyromonas*, *Capnocytophaga*, *Leptotrichia*, *Actinomyces*, *Corynebacterium*, *Rothia*, *Peptidiphaga*, *Kingella*, *Cardiobacterium*, *Streptococcus*, and *Abiotrophia*.   **Increases in Microbial Abundance**   - **Saliva Only:** An increase in the relative abundance of *Alloscardovia*, *Cryptobacterium*, and *Dialister* was noted in salivary samples. - **Subgingival Sites Only:** The subgingival biofilm exhibited an increase in the relative abundance of *Bacteroidetes*, *Olsenella*, *Lachnospiraceae, Filifactor*, *Peptostreptococcaceae*, and *Treponema*. | Increased Gingival Inflammation in vapers compared to non-smokers | No Comparison | Cotitine test, but no concentration and results not reported and self-reporting |
| Miluna-Meldere 2024 | No information provided | No information provided | Tannerella forsythia, Prevotella intermedia, and Porphyromonas gingivalis Increase in EC compared to NS | Nil | No Comparison | Self-reporting |
| Liu 2024 | No Changes | Decrease | **Increases in** Firmicutes and Fusobacteria, Veillonella and Neisseria, Leptotrichia,  **Decreases in** Porphyromonas Peptostreptococcus | Nil | TS caused greater microbial shifts | Self-reporting |

**NS:** Never Smokers
**TS:** Traditional Smokers
**EC:** Electronic Cigarette
**Perio:** Periodontitis
*Severe and moderate refer to periodontal status of participants

**Table S8: Risk of bias of cross-sectional studies**

| **Author** | **Year** | **Selection** | | | | **Comparability** | **Exposure** | | | **Total** |
| --- | --- | --- | --- | --- | --- | --- | --- | --- | --- | --- |
|  |  | *Case Definition* | *Representative  of cases* | *Selection of cases* | *Definition of controls* | Controlled for Cofounders | Ascertainment of exposure | Same method of ascertainment | Non-response rate same in both groups | Out of 9 |
| **Aldakheel** | **2020** |  | *** | *** | *** | * |  | * | * | 6 (Fair) |
| **Alzoubi** | **2020** | * | *** | *** | *** |  |  | * | * | 6 (Fair) |
| **Kiiun** | **2023** |  | *** | *** | *** | * |  | * | * | 6 (Fair) |
| **Stewart** | **2018** | * | *** | *** | *** | * (no perio) | * | * | * | 8 (Good) |
| **Wang** | **2022** |  | *** | *** | *** | *(no perio) |  | * | * | 6 (Fair) |
| **Park** | **2023** |  | *** | *** | *** | ** |  | * | * | 7 (Good) |
| **Tishchenko** | **2022** |  |  |  |  | No Perio |  | * | * | 2(Poor) |
| **Ganesan** | **2020** | * | *** | *** | *** | * |  | * | * | 7 (Good) |
| **Chopyk** | **2021** |  | *** | *** | *** | *(No perio) | * | * | * | 7 (Good) |
| **Liu** | **2024** |  | *** | *** | *** | * |  | * | * | 6 (Fair) |
| **Pushalkar** | **2020** | * | *** | *** | *** | ** |  | * | * | 8 (Good) |
| **Mokheem** | **2018** | * | *** | *** | *** |  |  | * | * | 6(Fair) |
| **Miluna-Meldere** | **2024** |  |  | *** | *** | (Perio included) |  | * | * | 5(fair) |
| **Chichonska** | **2021** | * | *** | *** | *** | * | * | * | * | 8 (Good) |
| **Yang** | **2023** | * |  | *** | *** | * (No Perio) | * | * | * | 7 (Good) |
| **Ying** | **2022** | * | *** | *** | *** | *(No Perio) |  | * | * | 7 (Good) |

**Table S9: Risk of bias of included cohort studies**

| **Author** | **Year** | **Selection** | | | | **Comparability** | **Exposure** | | | **Total** |
| --- | --- | --- | --- | --- | --- | --- | --- | --- | --- | --- |
|  |  | *Representativeness of exposed cases* | *Selection of non-exposed cases* | *Ascertainment of exposure* | *Demonstrated outcome of interest not present at beginning of studies* | Controlled for Cofounders | Ascertainment of exposure | Same method of ascertainment | Non-response rate same in both groups | Out of 9 |
| **Xu** | **2022** | *** | *** | *** | *** | * |  | * | * | 7 (Good) |
| **Thomas** | **2022** | *** | *** | *** | *** | * |  | * | * | **7 (Good)** |
